# Gut-brain Axis and migraine headache: a comprehensive review

**DOI:** 10.1186/s10194-020-1078-9

**Published:** 2020-02-13

**Authors:** Mahsa Arzani, Soodeh Razeghi Jahromi, Zeinab Ghorbani, Fahimeh Vahabizad, Paolo Martelletti, Amir Ghaemi, Simona Sacco, Mansoureh Togha, On behalf of the School of Advanced Studies of the European Headache Federation (EHF-SAS)

**Affiliations:** 1 Headache Department, Iranian Center of Neurological Research, Neuroscience Institute, Tehran University of Medical Sciences, Tehran, Iran; 2 Department of Clinical Nutrition and Dietetics, Faculty of Nutrition and Food Technology, Shahid Beheshti University of Medical Sciences, Tehran, Iran; 3 Cardiovascular Diseases Research Center, Department of Cardiology, Heshmat Hospital, School of Medicine, Guilan University of Medical Sciences, Rasht, Iran; 4 Headache Department, Neurology Ward, Sina University Hospital, School of Medicine, Tehran University of Medical Sciences, Tehran, Iran; 5 Department of Clinical and Molecular Medicine, Sapienza University of Rome, Rome, Italy; 6 Department of Virology, Pasteur Institute of Iran, Tehran, Iran; 7 Neuroscience section – Department of Applied Clinical Sciences and Biotechnology, University of L’Aquila, L’Aquila, Italy

## Abstract

The terminology “gut-brain axis “points out a bidirectional relationship between the GI system and the central nervous system (CNS). To date, several researches have shown that migraine is associated with some gastrointestinal (GI) disorders such as Helicobacter pylori (HP) infection, irritable bowel syndrome (IBS), and celiac disease (CD). The present review article aims to discuss the direct and indirect evidence suggesting relationships between migraine and the gut-brain axis. However, the mechanisms explaining how the gut and the brain may interact in patients with migraine are not entirely clear. Studies suggest that this interaction seems to be influenced by multiple factors such as inflammatory mediators (IL-1β, IL-6, IL-8, and TNF-α), gut microbiota profile, neuropeptides and serotonin pathway, stress hormones and nutritional substances. Neuropeptides including CGRP, SP, VIP, NPY are thought to have antimicrobial impact on a variety of the gut bacterial strains and thus speculated to be involved in the bidirectional relationship between the gut and the brain. According to the current knowledge, migraine headache in patients harboring HP might be improved following the bacteria eradication. Migraineurs with long headache history and high headache frequency have a higher chance of being diagnosed with IBS. IBS and migraine share some similarities and can alter gut microflora composition and thereby may affect the gut-brain axis and inflammatory status. Migraine has been also associated with CD and the condition should be searched particularly in patients with migraine with occipital and parieto-occipital calcification at brain neuroimaging. In those patients, gluten-free diet can also be effective in reducing migraine frequency. It has also been proposed that migraine may be improved by dietary approaches with beneficial effects on gut microbiota and gut-brain axis including appropriate consumption of fiber per day, adhering to a low glycemic index diet, supplementation with vitamin D, omega-3 and probiotics as well as weight loss dietary plans for overweight and obese patients.

## Introduction

Based on global burden of headache reports in 2016, it was estimated that approximately 14% of the adult population worldwide suffer from migraine [1]. The disease is three times more prevalent among females and imposes high burden at the individual and society level. According to Global Burden of Disease (GBD) study 2018, migraine has been recognized as the first leading cause of disability in those aged less than 50 years [2, 3]. The exact pathogenesis of migraine is still undefined but implies numerous factors, including the gut-brain axis [4].

**Fig. 1:**
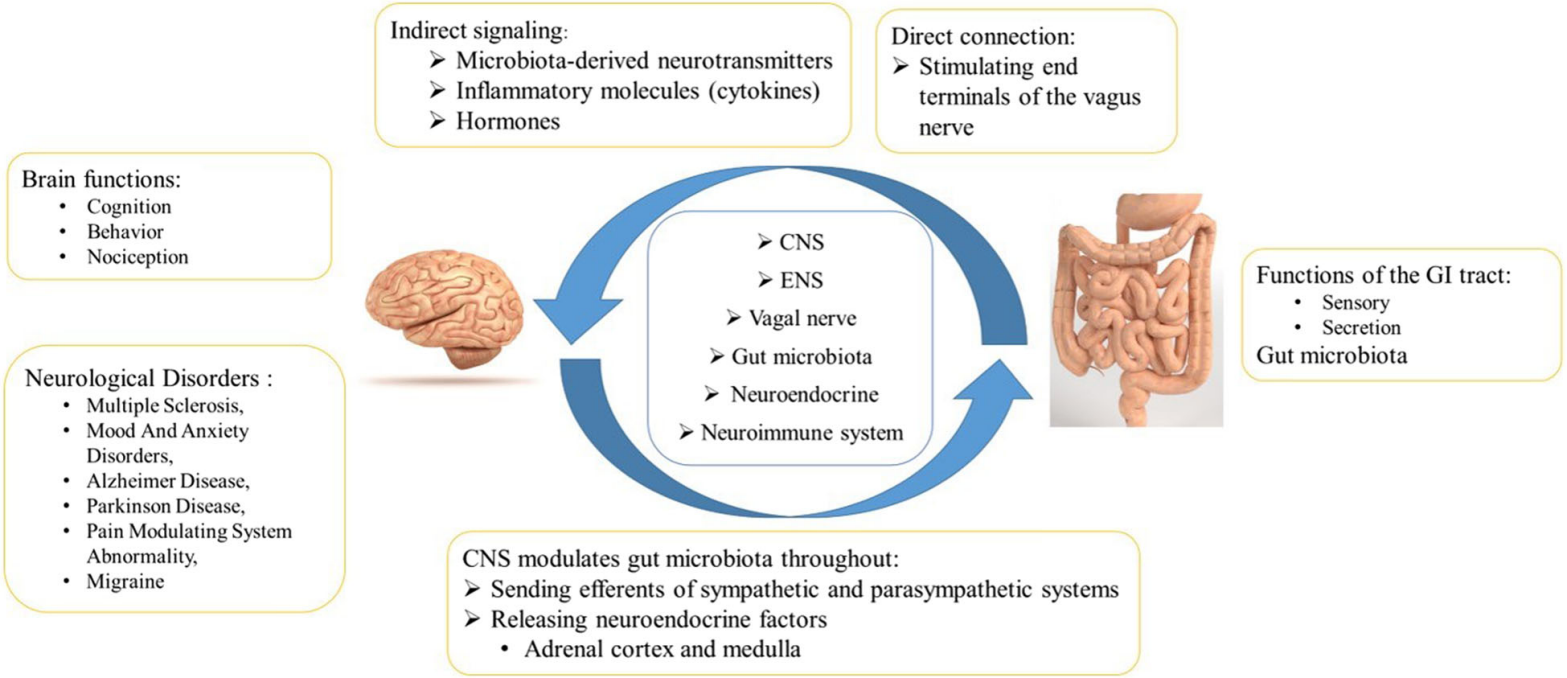
Gut -Brain Axis. CNS, Central nervous system; ENS, Enteric nervous system; GI, Gastrointestinal

The terminology “gut-brain axis “points out a bidirectional relationship between the GI system and the central nervous system (CNS). Brain normally regulates movements and functions of the GI tract (sensory and secretion). Hormonal factors through the hypothalamic pituitary adrenal (HPA) axis by mediating stress responses impact on the gut functions. On the other hand, GI system is believed to be able to affect the CNS. A number of the brain functions such as cognition, behavior and even nociception are under the influence of the gut system [5, 6]. The dysfunction of the gut-brain axis has been implicated in a number of neurological disorders such as multiple sclerosis, mood and anxiety disorders, Alzheimer disease, Parkinson disease, and migraine [5, 6]. Figure 1 depicts the mechanisms of the bidirectional relationship between the gut and the brain in migraine (Fig. 1). Several neurotransmitters have been supposed to play a role in this process including serotonin, dopamine, gamma-aminobutyric acid, and calcitonin gene-related peptide (CGRP) [6–8].

The present review article aims to discuss the direct and indirect evidence suggesting relationships between migraine and the gut-brain axis. As it will be clarified later, this interrelationship seems to be influenced by multiple factors such as inflammatory mediators, gut microbiota profile, neuropeptides, stress hormones and nutritional substances. In this regard, at first we will take a look at the involvement of inflammation in migraine headache and role of gut microbiome. Afterwards, the role of the neuropeptides specifically serotonin pathway in relation to migraine and gut-brain axis will be explored. Later, the current evidence on the association between migraine and gastrointestinal (GI) disorders including Helicobacter pylori (HP) infection, irritable bowel syndrome (IBS), celiac disease (CD) and inflammatory bowel disease (IBD) will be described. Finally, the effects of probiotic supplementation on migraine and possibly effective dietary approaches for migraine patients including modifying carbohydrate and fat intake, vitamin supplementation and weight loss diets will be discussed.

### The involvement of inflammation in migraine headache and role of gut microbiome

In the gut, immune cells and their inflammatory mediators such as interleukin (IL)-1β, IL-6 and IL-18, tumor necrosis factor alpha (TNF-훼), and interferon gamma (IFN)-훾 have been implicated as sensitizers of afferent endings and are known as inducers of visceral pain [9, 10]. Additionally, proinflammatory cytokines, including IL-1β, IL-6, IL-8, and TNF-α have been implicated in migraine pain and are increased during migraine attacks [11, 12].

Most of the studies aiming to investigate the roles of gut microflora in different disorders, usually use microbiota deprivation or “germ-free” murine models. These animals are grown in a sterile condition and therefore are microbiota-deficient [13]. In this regard, the importance of the immune system in the gut-brain axis and in migraine pathobiology is also supported by the evidence that hypernociception caused by inflammatory stimuli can decrease in germ-free compared to conventional mice [14]. This result highlights the important role played by gut microbiota in preparing the host adaptation to stress factors in the environment, which induce pain [14].

**Fig. 2:**
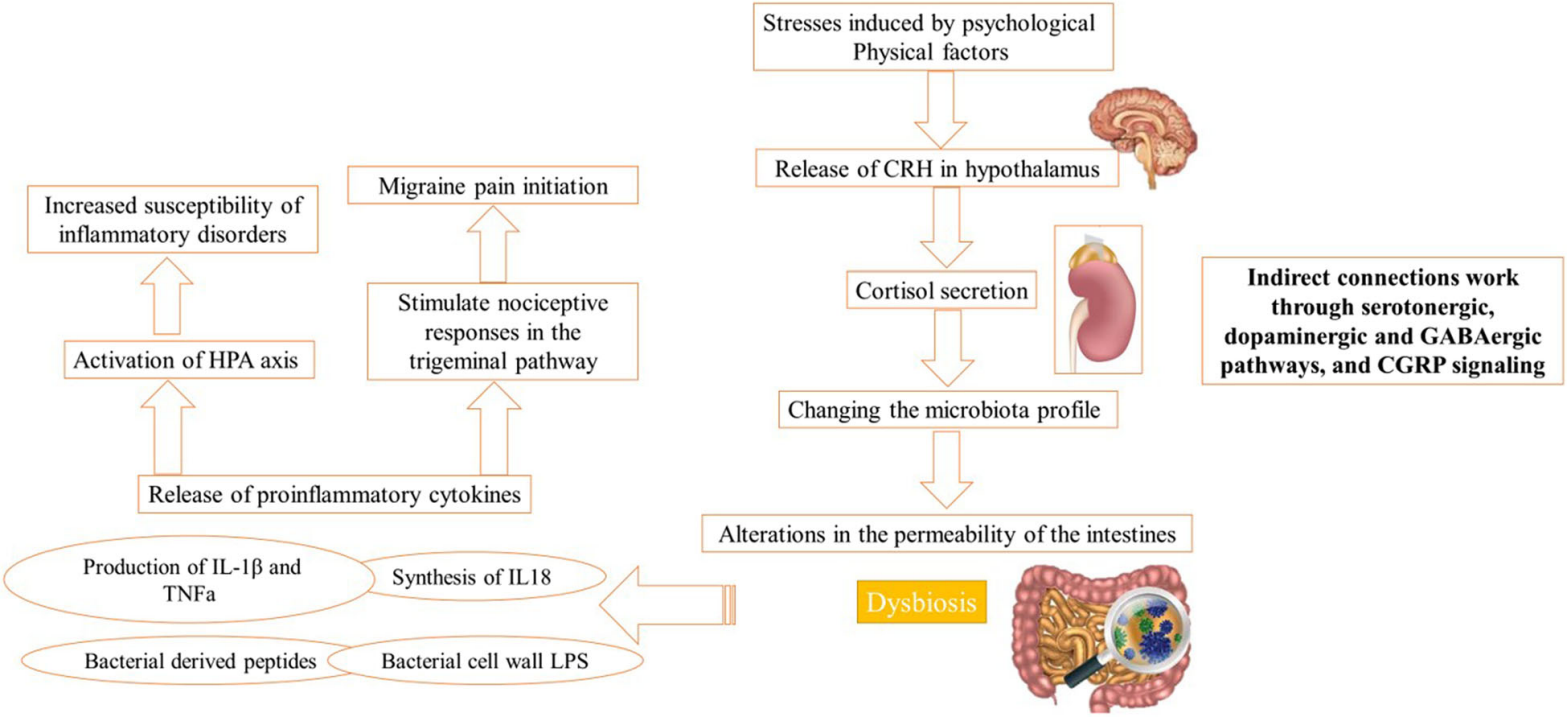
Mechanisms of the effects of gut bacteria in keeping normal balance of gut-brain axis via indirect signaling. CGRP, Calcitonin gene-related peptide; CRH, Corticotrophin-releasing hormone; HPA, Hypothalamic pituitary adrenal axis; LPS, lipopolysaccharides; IL, Interleukin; TNFa, Tumor necrosis factor alpha

It is noteworthy that the composition of the gut microbiota plays a major role in gut-brain axis. This happens via two mechanisms: indirect signaling, including microbiota-derived neurotransmitters, inflammatory molecules, and hormones; and direct connection with stimulating end terminals of the vagus nerve (Fig. 2). Also in this case, the mechanism is bidirectional as CNS can modulate gut microbiota throughout sympathetic and parasympathetic systems and by releasing neuroendocrine peptides [15]. Changes in the intestinal microbiota profile may occur as a result of psychological and physical stress factors. These stressors stimulate release of corticotrophin-releasing hormone in hypothalamus, that induces cortisol secretion from the adrenal glands, and may lead to alterations in the permeability of the intestines through changing the microbiota profile. Finally these events could lead to dysbiosis (changes in microbiota profile of the gut) [6, 16–19]. On the other hand, dysbiosis of GI microbiota and increased gut permeability may lead to activation of HPA axis through the release of proinflammatory cytokines such as IL-1β and TNF-훼. The release of cytokines may be inhibited by the stress-induced steroid response which anyhow increases the susceptibility of inflammatory disorders [20–23].

Substances such as CGRP, substance P (SP), vasoactive intestinal peptide (VIP), and neuropeptide Y (NPY), are thought to have antimicrobial impact on a variety of the gut bacterial strains (for instance *Escherichia coli*, *Enterococcus faecalis*, and Lactobacillus acidophilus) and thus speculated to be involved in the bidirectional relationship between the gut and the brain [15]. It has also been reported that the colonic concentration of SP increases in response to antibiotic treatment and subsequent dysbiosis while *Lactobacillus paracasei* administration attenuated this response [24]. Studies of peripherally administrated CGRP in animal models demonstrated CGRP inhibits basal and stimulated gastric acid secretion [25, 26]. In addition, CGRP is a potent inhibitor of pancreatic enzyme secretion via modulation of a central vagal out flow [27]. On the other hand, as CGRP signaling could be influenced by microbiota [15].

Nutritional factors may influence the mechanisms through which the gut microbiota manages gut health and immune function [28]. Short-chain fatty acids (SCFAs, namely: butyrate, propionate, and acetoacetate) are crucial in maintaining gut barrier integrity. These substances are produced by bacteria in the distal colon [29–32]. It has been suggested that gut microbiota activities and consequently the levels of SCFAs in the gut might be affected by dietary factors including fiber and probiotics [31]. Apart from affecting gut/systemic immunity, SCFAs bypass portal circulation and could reach the CNS via circulation. In CNS, SCFAs have neuroprotective properties. For instance, sodium butyrate, the salt of butyrate, stimulates cell proliferation and differentiation in the dentate gyrus, and enhances the expression of brain-derived neurotrophic factor (BDNF) and glial-derived neurotrophic factor (GDNF). Butyrate also shows anti-inflammatory effect in the brain by suppressing the synthesis of TNF-α, induced by the endotoxin lipopolysaccharides (LPS) through the suppression of nuclear factor κB [32]. Additionally, changes in SCFAs producing phyla can affect immune function of the host. Rapid and extreme dietary changes directly affect gut microbiota because they influence the beta diversity of the gut microbiota (scale of measuring the turnover of the microbiota species) [33, 34]. Interestingly, adding prebiotics (fermentable fibers) to high-fat diet was reported to restore the decreased levels of butyrate-producing bacteria and Bifidobacteria which also highlights the critical influence of diet on composition of the gut microbiota [35].

Therefore, due to the bidirectional association between permeability of the gut and inflammatory state, augmented gut permeability can stimulate inflammatory and immune responses through LPS leakage and subsequently proinflammatory cytokines can reinforce gut permeability [36, 37]. On the other hand, as mentioned, proinflammatory cytokines such as TNF-α, IL-1β and IL-6 could also affect nociceptive responses in the trigeminal pathway and play a role in migraine pain initiation [38–43]. Figure 2 demonstrates the mechanisms of the effects of gut bacteria in keeping normal balance of gut-brain axis via indirect signaling in which these inflammatory cytokines play an important role.

#### The role of the neuropeptides in migraine headache with a focus on the gut-brain axis

Glutamate, as an excitatory neurotransmitter, plays a role in migraine pathophysiology through different effects including cortical spreading depression (CSD), central sensitization, and by stimulating trigeminovascular system. Increased glutamate levels in the plasma and CSF of migraineurs in comparison with control subjects have been reported previously [44, 45]. On the other hand, the role of this neurotransmitter in the enteric nervous system (ENS) and the gut-brain axis (transmitted along afferent neurons from the gut into the brain) has been studied in depth. Also, it was indicated that glutamate might affect the inflammation and oxidative stress in the GI tract [46]. The disturbances in glutamate pathway appeared to be involved in the pathogenesis of a variety of GI disorders such as IBD, IBS, gastroesophageal reflux, gastric acid hyper-secretory disorder [46].

NPY, a probable indicator of noradrenergic system function, affects cerebral blood flow through cerebral circulation regulation. It is worth mentioning that higher levels of NPY has been detected in ictal phases of migraineurs [15, 44]. Interestingly, this neurotransmitter has been detected at all levels of the gut-brain axis. NPY pathway is also assumed to contribute to changing GI function and its blood flow, immunological system and inflammation status, pain, homeostasis of energy, emotion, mood, as well as behavior and other functions of the brain (e.g. cognition) [15, 44, 47].

One of the main biomarkers of migraine, CGRP, inhibits gastric acid secretion and may suppress food intake [48]. As mentioned, CGRP signaling could be influenced by microbiota as such increased level of this neurotransmitter in dysbiosis might be one of the probable justification for prescribing probiotics in migraine [15].

Cholecystokinin (CCK) is synthesized by enteroendocrine cells in the mucosal lining of small intestine (I cells), mammalian brain (eg. cortex, thalamus, mesolimbic, periaquiductal gray matter (PAG) and midbrain), and spinal cord. This peptide inhibits gastric emptying and acid secretion, stimulates gall bladder contraction and pancreatic secretion, and provokes satiety feeling in the brain via CCK1 receptor. The presence of CCK1R on vagal afferent terminals lying in the wall of GI tract further confirms the hypothesis of endocrine and paracrine collaboration. The location of CCK1R in mesolimbic structures, hypothalamus and brain stem nucleus and its interaction with dopamine, serotonin, glutamate, hypothalamic hormones, and neuropeptides explains specific roles of CCK in behavior, mood, and extrapyramidal function [49, 50]. Like CGRP, CCK is produced in the PAG and may be responsible for endogenous pain signaling system and its level increases in migraine [51]. CCK is also present in trigeminal ganglion, such that stimulation of trigeminal ganglion results in increasing local CCK [52]. On the other hand, obese patients had a significantly higher frequency and severity of migraine attacks as compared to overweight or normal weight individuals [53]. A meta-analysis study found an increased risk of having migraine in obese subjects; also the risk of chronic migraine is higher in obese subjects than in normal weight group [54]. One of the possible explanations for the association between migraine and obesity is CCK secretion in response to high fat diets as intra-duodenal free fatty acids stimulate CCK secretion [55].

#### Serotonin pathway involvement in migraine headache with a focus on the gut-brain axis

It has been proposed that tryptophan-kynurenine pathway might be one of the main ways by which GI microbiota may affect the function of the CNS due to the presence of synthetic enzymes in bacterial strains of the intestine that may produce tryptophan metabolites (such as quinolinic and kynurenic acids) [16, 56]. As tryptophan is the precursor of serotonin, the amount of this neurotransmitter in the brain depends on hthe levels of this amino acid [16, 56].

The role of gut microbiota in anxiety, depression and the HPA axis has been studied through assessing the effects of prebiotics and probiotics administration in these conditions [57–59]. It has been reported that “germ-free” mice may have increased plasma levels of serotonin and tryptophan. These reports may justify the role played by hormonal factors in the association between gut bacterial strains and tryptophan levels [16, 56, 60]. Moreover, the turn-over of serotonin as shown by increased levels of the pathway metabolites, has been reported to be elevated in germ-free animals; while it has been demonstrated that supplementation with probiotic lead to lessen the concentration of 5-hydroxyindoleacetic acid (5-HIAA) and kynurenine in the cortical frontal lobe. These effects might be attributed to the impact of the gut microbiota on the enzymes affecting availability of tryptophan and serotonin pathway and the amino acid utilization by the bacteria [61]; however, the exact mechanisms and the role of different factors including gender, and strains of mice have not been elucidated yet.

Moreover, the presence of serotonin receptors on immune cells including monocytes, macrophages, lymphocytes, and dendritic cells, may shed light on the impact of this neurotransmitter on immune modulation. This issue might also explain effects of serotonin on inflammatory state of the intestine and its role in increasing the susceptibility for GI diseases (such as Crohn’s disease, ulcerative colitis, celiac disease and diverticulitis) [61].

### Migraine and gastrointestinal disorders

To date, several researches have shown that migraine is associated with some GI disturbances such as diarrhea, constipation [62, 63], dyspepsia [64], and gastroesophageal reflux (GERD) [65]. Additionally, some GI disorders including HP infection [66, 67], IBS [68, 69], CD [70], and IBD [71–73] have also been associated with migraine.

### Helicobacter pylori infection

Based on the findings of a meta-analysis of 5 case-control studies, about 45% of migraineurs harbor HP, while the prevalence rate among healthy controls was estimated at about 33% [66]. It is noteworthy that when aiming at investigating the role of HP infection in headache pathogenesis, various strains of the bacterium, ethnicity of the studied patients, the variation of HP in different regions, and the potential pathological differences which would be present in different subtypes of headache, should be considered [18].

According to a review article findings, evidence supported that HP eradication may be associated with relief of migraine symptoms [67]. Also, Faraji et al. 2012 [74] compared the effects of HP eradication treatment with placebo among patients who received migraine treatment within a randomized, double-blind, controlled trial. They reported that at the end of study, patients who received HP eradication treatment showed lower migraine-related disability level compared with those in placebo group [74].

HP infection is supposed to be related to a persistent chronic inflammatory state, which in turn may lead to increased production of inflammatory mediators and vasoactive compounds [18, 75]. Therefore, the proposed mechanism for the association between migraine and HP infection may include induced immune, inflammatory, and vascular responses and subsequent release of immune cells, inflammatory and vasoactive agents into the gastric mucosa that may finally lead to hypersensitivity of brain pain-sensitive structures [66]. Moreover, the neuroendocrine release of other factors involved in migraine pathophysiology including serotonin, SP, and VIP appeared to be altered by the inflammatory state caused by the bacterium [66]. Particularly, it has been demonstrated that the levels of CGRP were elevated in HP-induced duodenal ulcers when comparing to healthy individuals [18, 44, 76]. Furthermore, higher IL-10 plasma levels have been observed during migraine attacks [77]; on the other hand, some studies showed that HP infection is associated with increased levels of IL-12 and IL-10 [78–80]. These observations may suggest that IL-10 stimulated by HP may exacerbate the severity of migraine.

Collectively available data indicate that HP may impact on migraine symptoms. However, at the moment this is still a matter of research and there is no evidence to support systematic search of the infection in migraineurs nor the treatment of the infection specifically aimed to improve migraine symptoms.

### Irritable bowel syndrome

IBS and migraine share some similarities, i.e. both disorders are chronic, recurrent [69], and more prevalent among women, have high individual/social burden, highly affect the patients’ quality of life, and seem to be accompanied by a number of comorbid psychological diseases especially anxiety [69, 81–85]. Furthermore, central, visceral and thermal cutaneous hypersensitization are common among both disorders [69]. Overall, 60% of migraineurs have allodynia and IBS patients reported to have allodynia aside from visceral hypersensitivity [69]. N-methyl-D-aspartate (NMDA) may probably be responsible for allodynia among the IBS patients [86].

There is an established association between migraine and IBS, such that IBS was revealed to be common among migraineurs and migraine was reported to be prevalent among IBS suffers [18]. A prevalence cohort study showed that in comparison to non-IBS subjects, IBS patients had a 40–80% higher prevalence odds of migraine, depression, and fibromyalgia [68]. A further study, found that about 17% of IBS patients had migraine while only 8% of the control group suffered from this type of headache [87]. A meta-analysis on 6 studies showed that individuals who suffered from IBS had coexisting headache with an estimated OR of approximately 2.7 [69]. On the other hand, higher prevalence of IBS in migraineurs has been also reported with a rate ranging from 4% to 40% [69, 81–85]. Migraineurs with long headache history and high headache frequency had a higher chance of being diagnosed with IBS [88].

Mechanisms underlying the association are not entirely clear. Similar to migraine, IBS can alter gut microflora composition and thereby may affect the gut-brain axis and inflammatory status [13, 89–93]. Moreover, food allergies/intolerances that seem to cause migraine attack initiation and relapse of IBS may also explain the association between the disorders [92]. With respect to this, food elimination diets based on IgG antibodies may effectively reduce symptoms in migraine patients with concomitant IBS [94]. Hereditary and genetic polymorphism might explain at least in part the comorbidity between migraine and IBS [69].

Also 5H-T has been postulated to play a role in the association of migraine and IBS. Serotonin release from entrochromaffin cells in the gastric pits of the stomach luminal epithelium stimulates gastric acid secretion, sensory and motor GI reflexes which in turn activates the ENS [69, 95, 96]. Interestingly, it was observed that patients with IBS had higher systemic levels of serotonin and kynurenic acid when compared to healthy individuals, which implies that serotonin function in these patients might be impaired as a result of augmented kynurenic pathway activation [18, 61]. Therapeutic agents modulating serotonin receptors are effective in patients with both disorders (i.e. IBS and migraine) [97]. Sexual hormones are also believed to play a role, because migraine and IBS are more prevalent among females. In addition, estrogen enhances serotonin release in the brain and increases pain sensitivity [98].

### Celiac disease

CD is an autoimmune multisystem condition caused by gluten peptide in genetically susceptible individuals [99]. According to the available data, the prevalence of CD is 1.4% [100]. A variety of neurologic manifestations have been observed with CD such as epilepsy, ataxia, cerebellar ataxia, mood disorders, encephalitis, peripheral neuropathy, neuromuscular disorders, dementia, learning disorders, developmental delay and migraine [101]. A “nutritional-microbial-epithelial-neuronal” akin to “environmental-luminal-mucosal-neuronal” brain network may be responsible for these extra-intestinal manifestations [102]. Studies have shown that patients with CD have higher prevalence of migraine compared with healthy controls and vice versa [103, 104]. It is estimated that about 21–28% of patients with CD have migraine [104–106]. Migraine-like headache may represent the initial feature of CD [103, 107–109]. Some studies suggest that evidence of occipital and parieto-occipital calcifications and white matter abnormalities at brain neuroimaging are associated with comorbid CD in subjects with migraine [103, 110–112]. Clinicians may consider to search for CD in subjects with migraine in the presence of such alterations especially if other symptoms suggestive of CD are present. The association between migraine and CD may be attributed to several concurrent mechanisms including proinflammatory cytokines induced by gluten (e.g. IFN-훾 and TNF-훼 that are thought to enhance CGRP levels), lack of vitamins and macro elements due to malabsorption, vascular tone disturbances, nervous system hypersensitivity, brain hypoperfusion and perivascular inflammation [18, 103, 113–117]. It was also suggested that CD treatment may improve headache [118]. There are some studies which suggest that institution of gluten-free diet also may be effective in decrease of migraine frequencies [18, 103, 119].

### Inflammatory bowel disease

IBD is a chronic relapsing-remitting inflammatory condition that consists of Crohn disease and ulcerative colitis [120]. Data about possible relationship between headache or migraine and IBD are scarce [19]. A case-control study showed that the prevalence of headache was higher in IBD individuals compared to control group (46% vs 7%) [71]. In a Brazilian study, headache was the most common neurologic manifestation in IBD patients; 25% of patients with headache fulfilled the criteria for migraine [72]. A cross-sectional study performed in a tertiary-care headache center demonstrated that migraine prevalence was two-fold higher in IBD individuals compared to general population [73]. A further study reported a higher prevalence of migraine in patients with IBD (21.3%) compared to non-IBD subjects (8.8%) [121]. Although the exact mechanism remained unclear, but autoimmune-inflammatory responses, malabsorption, endothelial dysfunction that are present in IBD as well as immunosuppressive treatments prescribed for the disorder might be involved in pathophysiology underlying the association between IBD and migraine [121–123].

### Dietary approaches in migraine headache with a focus on the gut-brain Axis

Different dietary approaches have been suggested for subjects with migraine [117], but at the moment it is unclear if any diet can be used to improve migraine management. It is important that those studies are very difficult to put into context because difficulties in maintaining the dietary strategy, in establishing adherence to the regimen, and in blindness in the study design. Overall, there is lack of high quality, well-designed clinical trials in this field, and available data are preliminary and should be interpreted with caution.

#### Probiotic supplementation

Probiotic supplementation may modulate migraine attacks. Possible mechanisms of action are unclear and include promoting SCFA production in the gut and improving epithelial integrity of the intestine and enhancing inflammation by nuclear factor kappa-B (NF-κB) pathway suppression and therefore lowering proinflammatory cytokines levels [37, 89, 90, 124–127]. Probiotics could also enhance the gastric emptying rate and attenuate gastric stasis - a common GI complaint among migraineurs - by neuroimmune interaction [91, 128, 129].

Some studies have explored the beneficial effects of probiotic administration on migraine headaches [89, 126, 127, 130]. In a randomized double-blind controlled trial, the effect of daily adminstration of a 14-strain-probiotic mixture or placebo for 8 weeks in chronic and 10 weeks in episodic migraineurs was evaluated. Probiotic administration resulted in significant improvements in frequency and severity of migraine and the consumption of abortive medications in the studied population despite no significant changes in serum levels of selected inflammatory biomarkers [127].

In an open-label trial in 40 migraineurs, 12-week supplementation with probiotic + minerals + vitamins + herbs, resulted in significant improvement in patients quality of life in approximately 80% of the subjects and pain relief in more than half of the migraineurs [126]. In a further study, supplementation with a probiotic mixture of 7 bacterial strains reduced the migraine attacks frequency by about a quarter and also lowered migraine-related disability [89]. However, a more recent study by the same group of authors reported conflicting results and found no changes in migraine-related outcomes with the same treatment [93]. The studied that were performed on effects of probiotic supplementation on migraine headache are summarized in Table 1.

**Table 1:** Literatures on effects of probiotic supplementation on migraine headache

authors and year	Type of article	Sample size	Type of probiotics	Duration of treatment	Results
De Roos N, Giezenaar C, Rovers J, et al. 2015 [89]	Clinical trial	29 patients	2 g/d of a probiotic food supplement (Ecologic(®)Barrier, 2.5 × 10(9) cfu/g)	12 weeks	1) number of migraine days/month decreased significantly 2) The MIDAS score improved 3) Headache Disability Inventory (HDI) did not change significantly
de Roos N, van Hemert S, Rovers J, et al. 2017 [130]	Randomized control trial	63 patients (probiotic (*n* = 31) placebo group (*n* = 32))	multispecies probiotic (5 × 10^9^ colony-forming units) or placebo daily	12 weeks	No significant benefit from a multispecies probiotic compared to a placebo on the outcome parameters of migraine and intestinal integrity
James Sensenig N, Jeffrey Marrongelle D and CCN MJ S. T. 2001 [126]	Clinical trial	40 patients	Two nutritional formulations contained probiotics + minerals + vitamins + herbs	3 months	80% of the participants experienced significant improvements in quality of life and pain relief in more than half of the migraineurs
Martami F, Togha M, Seifishahpar M, et al. 2019 [127]	randomized double-blind controlled trial	40 episodic and 39 chronic migraine patients	14-strain probiotic mixture or placebo	10 weeks	Significant reduction in migraine attacks, migraine severity, and the number of abortive drugs in the probiotic group compare to the placebo group

#### Carbohydrate

Gut microbiota fermented resistance carbohydrate to different metabolites, such as SCFAs [131]. The dietary shift to high resistance polysaccharides increases SCFAs levels [28]. In a randomized clinical study, 350 migraineurs were allocated to low glycemic index diet (considered as a high-fiber intake) group or to prophylactic medications group (either propranolol, flunarazine, amitriptyline or topiramate) in a 1:1 ratio. One month after dietary restrictions, the frequency of attacks was significantly reduced in both the diet and the pharmacological group. The befit was maintained at 90-day. Severity of attacks was reduced at both 30- and 90-day in the drug group but only at 90-day in the diet group [132].

#### Fat

Chronic exposure to omega-3 in utero and early life, increases the proliferation of Lactobacillus and Bifidobacterium and sp., results in more SCFAs production [133]. Four studies assessed the effect of low-fat diets in migraine prophylaxis. Reducing dietary fat intake for three months resulted in the reduction in headache intensity, frequency and abortive medicine consumption [53, 134–136]. In a 12-week trial adults with chronic migraine randomly assigned to have high omega-3/low omega-6 diet or low omega-6 diet. Individuals on high omega-3/low omega-6 diet experienced higher improvement in their headache compared to migraineurs on low omega-6 diet [134]. The observed effect was attributed to the followings [117]: [1] the balance between the two eicosanoid pathways, omega-6 and omega-3, contribute to inflammation control [2]; omega-6 fatty acids promote vasodilation [3]; high-fat diet induces hypercoagulability [4]; dietary fat affects serotonin release from platelet. Although high-fat diet reduces the proliferation of the SCFAs producing bacteria, the effect of fat intake on gut microflora and SCFAs production in migraineurs was not addressed yet in the studies. Table 2 represents a summary of the studies were conducted on effects of low fat diet on migraine headache.

**Table 2:** Literatures on effects of low fat diet on migraine headache

Authors and year	Type of article	Sample size	Type of diet	Duration of treatment	Results
Ferrara LA, Pacioni D, Di Fronzo V, Russo BF, Speranza E, Carlino V, et al. 2015 [53]	Crossover intervention trial	83 episodic or chronic migraineurs (63 female and 20 male)	a low-lipid and a normal-lipid diet	3 months + 3 months	Significant reduction in severity and number of migraine attacks
Ramsden CE, Zamora D, Faurot KR, et al. 2013	randomized trial	67 patients with chronic daily headache (H3-L6 = 33, L6 = 34)	High omega-3 + low omega-6 fatty acid (H3-L6) or low omega-6 fatty acid (L6)	12 weeks	Significant reduction in HIT score, headaches day per month, and headache hours per day in H3-L6 group
Bic Z, Blix GG, Hopp HP, Leslie FM, Schell MJ. 1999 [135]	Clinical trial	54 migraineurs	limit fat intake to no more than 20 g/day	12 weeks	Significant decrease in headache frequency, intensity, and duration and medication intake
Bunner AE, Agarwal U, Gonzales JF, Valente F, Barnard ND. 2014	Crossover trial	42 migraineurs	dietary instruction (a low-fat vegan diet) and placebo supplement	36 weeks (16 weeks + 4 weeks washout+ 16 weeks)	Significant reduction in headache severity and frequency during the diet period

### Vitamins

Vitamin D3 supplementation affected the intestinal microbiota composition. Supplementing healthy individuals with vitamin D3 for 8 weeks also significantly reduced Helicobacter sp. count [137]. Several studies reported that serum levels of vitamin D might be associated with increased risk of migraine/headache [138, 139]. Further, it has been suggested that the prevalence of deficiency/insufficiency of this vitamin may be higher in patients who suffer from migraine/ headache when comparing to headache-free individuals [139]. Also favorable effects of vitamin D supplementation on intensity and frequency of migraine attacks have been reported [139].

### Weight loss approaches in migraine/headache

Animal studies showed that obesity reduced gut permeability, reduced expression of tight junctions and could influence intestinal microbiota composition [140]. Pieces of evidence showed that obesity could increase the risk of episodic and chronic migraine [54, 141] while weight reduction can decrease the intensity, frequency, and duration of migraine headache in adults [142–144] and adolescents [145]. The link between obesity and headache was proposed to be attributed to shared pathophysiological features. Evidence showed an increase in CGRP plasma level of adult with obesity which is also pointed out in patients with migraine [146]. Furthermore, a rise in proinflammatory cytokines, such as IL-6 and TNF-훼 was reported in obese individuals and at the acute headache onset [147]. A summary of the studies on effects of weight loss on migraine headache is shown in Table 3.

**Table 3:** Literatures on effects of weight loss on migraine headache

authors and year	Type of article	Sample size	Type of intervention	Duration of treatment	Results
Bond DS, Vithiananthan S, Nash JM, Thomas JG, Wing RR. 2011 [142]	Prospected observational study	24 migraineurs	Bariatric surgery	6 months	Significant reduction in headache severity, and headache-related disability
Novack V, Fuchs L, Lantsberg L, Kama S, Lahoud U, Horev A, et al. 2011 [143]	Prospective study	29 premenopausal obese women with diagnosis of migraine	Bariatric surgery	6 months	Significant reduction in frequency of migraine attacks, duration of the attacks, and medication use during the attack and improvement of headache-related disability post bariatric surgery
Verrotti A, Agostinelli S, D’Egidio C, Di Fonzo A, Carotenuto M, Parisi P, et al. 2013 [145]	Clinical trial	135 obese adolescent migraineurs	dietary education, specific physical training, and behavioral treatment	12 months	Significant decrease in headache frequency and intensity, use of acute medications, and disability

## Conclusion

The current evidence shows that the gut-brain axis may impact on migraine despite the mechanism explaining this interaction is not entirely clear. Generally, this interaction seems to be influenced by multiple factors such as inflammatory mediators (IL-1β, IL-6, IL-8, and TNF-α), gut microbiota profile, neuropeptides and serotonin pathway, stress hormones and nutritional substances. Neuropeptides including CGRP, SP, VIP, NPY are thought to have antimicrobial impact on a variety of the gut bacterial strains and thus speculated to be involved in the bidirectional relationship between the gut and the brain. Additionally, there is comorbidity between migraine and a number of conditions including HP infection, IBS, IBD, and CD. According to the current knowledge, migraine headache in patients harboring HP might be improved following the bacteria eradication. Migraineurs with long headache history and high headache frequency have a higher chance of being diagnosed with IBS. IBS and migraine share some similarities and can alter gut microflora composition and thereby may affect the gut-brain axis and inflammatory status. Migraine has been also associated with CD and the condition should be searched particularly in patients with migraine with occipital and parieto-occipital calcification at brain neuroimaging. In those patients, gluten-free diet can also be effective in reducing migraine frequency. Diet strategies may impact on migraine course and could represent a valuable instrument to improve migraine management. However, no definite conclusion can be drawn because of the limited evidence on migraine management with diet. It can be hypothesized that prescribing dietary approaches with beneficial effects on gut microbiota and gut-brain axis including appropriate consumption of fiber per day, adhering to a low glycemic index diet, supplementation with vitamin D, omega-3 and probiotics as well as weight loss dietary plans (in case of obese patients) could lead to improvements in migraine associated features.

## Data Availability

All included references in the present review article are available on the Internet.

## Abbreviations

5-HIAA: 5-hydroxyindoleacetic acid
BDNF: brain-derived neurotrophic factor
CCK: cholecystokinin
CD: celiac disease
CGRP: Calcitonin-Gene-Related-Peptide
CM: chronic migraine
CNS: central nervous system
CRH: corticotrophin-releasing hormone
CSD: cortical spreading depression
EM: episodic migraine
ENS: enteric nervous system
GBD: Global Burden of Disease
GDNF: glial-derived neurotrophic factor
HP: Helicobacter pylori
HPA axis: The hypothalamic pituitary adrenal axis
IBD: Irritable Bowel Syndrome
IBS: Irritable Bowel Syndrome
ICHD-3: International Classification of Headache Disorders 3
IFN-훾: interferon gamma
IL: interleukin
LPS: lipopolysaccharides
MDA: malondialdehyde
NF-κB: nuclear factor kappa-B
NMDA: N-methyl-D-aspartate
NO: nitric oxide
NPY: Neuropeptide Y
NSAIDs: nonsteroidal anti-inflammatory drug
PAG: periaquiductal gray matter
PUFA: Polyunsaturated fatty acid
ROS: Reactive Oxygen Sepsis
SCFAs: short-chain fatty acids
SP: substance P
TNF-α: tumor necrosis factor-α
VAS: visual analogue scale
VIP: vasoactive intestinal peptide
